# E-learning for research capacity strengthening in sexual and reproductive health: the experience of the Geneva Foundation for Medical Education and Research and the Department of Reproductive Health and Research, World Health Organization

**DOI:** 10.1186/s12960-016-0173-0

**Published:** 2016-12-07

**Authors:** Karim Abawi, Venkatraman Chandra-Mouli, Igor Toskin, Mario Philip Festin, Lynn Gertiser, Raqibat Idris, Hanan Hamamy, Moazzam Ali, Ameyo Masakhwe Bonventure, Marleen Temmerman, Aldo Campana

**Affiliations:** 1 Geneva Foundation for Medical Education and Research, Geneva, Switzerland; 2 Department of Reproductive Health and Research, World Health Organization, Geneva, Switzerland; 3 Department of Genetics, University of Geneva, Geneva, Switzerland

## Abstract

**Abstract:**

Technological advancement has resulted in the increasing use of e-learning and online education, initially in high-income countries and increasingly in low- and middle-income countries.

**Background:**

In 2010, the Geneva Foundation for Medical Education and Research, in collaboration with the World Health Organization and partner institutions, developed an online postgraduate course “From Research to Practice: Training Course in Sexual and Reproductive Health Research”. This course takes advantage of the advancing Internet technology to provide training opportunities to health professionals mostly from low- and middle-income countries whose access to quality education is constrained by time, financial resources, or both.

**Case presentation:**

To assess the outcomes of the course, an evaluation was conducted by sending a self-administered questionnaire to graduates of the 2010–2012 programme. The objectives were to determine if the graduates had applied the knowledge gained from the course to their work and whether they had implemented their research project developed during the course. The evaluation also appraised the number of graduates who participated in the design or implementation of a new research project since the course concluded and whether the course had contributed to advancement in their careers. A total of 175 of 219 course graduates answered the questionnaire.

The evaluation revealed that the majority of respondents (98%) had utilized the knowledge acquired, with nearly half of them (47%) having published a scientific paper as author or co-author. About a third of respondents (39%) had implemented their course research project and about three quarters of them (74%) have been involved in the design or implementation of a research project after completing the course. Over three quarters (81%) of respondents opined that the course had contributed to their career advancement and almost half of them (46%) had a career promotion as a direct or indirect benefit of the course.

**Conclusion:**

We surmise that the course positively impacted the participants’ knowledge and understanding of sexual and reproductive health, which they applied in their professional work, as well as strengthened their research capacity. Success factors for the e-learning programme include tailor-made content to meet participants’ needs, flexibility of access, and ongoing engagement/personal interactivity with course coaches.

## Introduction

E-learning is increasingly being used worldwide in a number of contexts. It provides a useful tool to improve the abilities of personnel involved in preventive and curative health services worldwide, thereby addressing the increasing inequalities of medical care both within and between countries. Because of its flexibility, low cost, and ease of updating, many medical education programmes incorporate e-learning approaches. Moreover, e-learning is more practical in continuing medical education, which is an indispensable part of professional life for physicians and other health service providers. It has the advantage of providing health professionals with the opportunity to manage the timing of education themselves [1–3].

According to the latest World Health Organization (WHO) report, 57 countries lack an estimated 4.2 million healthcare providers (medical doctors, nurses, and allied healthcare workers). In response to this penury, many countries have made efforts to invest more in developing human resources and equitably distributing it, with a special focus on underserved areas. The increase in the number of training institutions reveals the challenge of quality education, as in the majority of low- and middle-income countries there exists a shortage of qualified teaching faculty with up-to-date knowledge. In this situation, e-learning can provide an opportunity to extend faculty availability to health professionals from low- and middle-income countries [4, 5].

This paper presents the outcome evaluation of the online course, which is provided yearly by the Geneva Foundation for Medical Education and Research (GFMER) and its partners to health professionals from different countries. This course was designed to leverage the advancements in online learning in order to strengthen the proficiency of healthcare providers through dissemination of current guidelines and bolster the number of healthcare providers in low-income countries.

The objectives of the evaluation were to determine the following:

- Whether participants applied their learning in their work, tested or implemented the research project they developed as part of the course, were involved in developing a new research project following the course, and published a scientific paper following the course; and
- Whether participation in the course helped them advance in their careers

An additional objective was to determine whether the approaches used by GFMER attained an acceptable attrition rate.

## Background

From 2003 to 2009, the Geneva Foundation for Medical Education and Research (GFMER), in partnership with the World Health Organization (WHO) and other partner institutions, organized a face-to-face 1-month intensive course on sexual and reproductive health research at the WHO headquarters in Geneva. The course focused on research methodology and attracted health professionals from around the world, particularly from developing countries.

Since 2010, in order to reach more health professionals, GFMER, in collaboration with the WHO and other partners, has developed an 8-month online course on sexual and reproductive health research: “From Research to Practice: Training Course in Sexual and Reproductive Health Research”, specially developed for health professionals, whose access to learning is constrained by time, financial resource, or other constraints and for whom access to quality education and learning is limited.

## Case presentation

The intended participants of this course are mature health professionals involved in clinical and public health work, particularly in the field of sexual and reproductive health (medical doctors, nurses, midwives, social scientists, etc.), with a focus on developing countries.

The Foundation has different strategies for recruiting eligible candidates for this course, such as course announcements on GFMER’s website and promotion by GFMER country coordinators, GFMER members, and partner institutions in different countries (universities, WHO collaborating centres, and non-government organizations (NGOs)).

The contents of the course are based on WHO up-to-date guidelines that reflect that reality of the situation in terms of sexual and reproductive health particularly in low- and middle-income countries and tailored to meet the needs of a variety of learners with different levels of education and living and working in different settings. The course consists of five specific modules: Maternal and Perinatal Health, Sexually Transmitted Infections and HIV/AIDS, Family Planning, Adolescent Sexual and Reproductive Health, and Community Genetics. Two additional modules on research methodology and sexual and reproductive rights support the above-mentioned modules. The content of each module is based on the latest knowledge and current practical experience in order to address relevant needs and challenges in the field of sexual and reproductive health research and implementation. Each module is coordinated by an expert who determines the content of the given module and assigns a facilitator to cover each of the topics presented in the module.

The facilitators of each module have experience in giving online lectures and presentations and in conducting conferences. The majority of them are highly qualified professionals from WHO, universities, and specialized agencies. A total of 36 experts in their field from the WHO, GFMER, and other national and international institutions are involved in teaching and tutoring for the online training course. Moreover, the GFMER has appointed a total of 18 coordinators in Afghanistan, Bhutan, Bolivia, Brazil, Ecuador, Egypt, Ethiopia, India, Iran, Kenya, Macedonia, Mexico, Mongolia, Mozambique, Nigeria, Sudan, Turkey, and Uganda. The coordinators are from universities, health ministries, hospitals, and NGOs. The majority of them are former students of GFMER course. The main task of a country coordinator is student coaching at the local level, i.e. helping the students access the teaching materials and answering questions they might have. As the majority of students’ paperwork (assignments and research protocol) is based on the analysis of local situations in regard to sexual and reproductive health needs and problems, local coordinators can offer the appropriate assistance to the students. GFMER teaching staff and former students are assigned to those participants who come from countries where the Foundation does not have a coordinator.

The course teaching methods consist of online lectures (recorded, didactic presentations), key reading materials, additional references, and referrals to related websites. Since the students come from many different countries and time zones, organizing real-time sessions is not appropriate. For this reason, we limit them throughout the course.

In order to successfully complete a module, participants need to complete a written assignment, which requires them to apply technical knowledge about sexual and reproductive health in a real-life context. The aim is for them to become more familiar with scientific reading and writing and to use the knowledge acquired in the training course in their day-to-day professional practice.

GFMER has created an online community, using Google Groups, Facebook, and Twitter, for the training course in order to improve communication with and between participants and to provide access to academic and administrative support. The diversity among the participants and the network facilitated by GFMER provide an opportunity to learn from and share experiences with each other.

At the end of the course, the participants receive a certificate if they meet the following conditions: (1) completion and timely submission of assignments for the course modules and (2) development of a research protocol on a topic relevant to their practice, which is approved by the tutors.

### Methodology to study the impact of the course

A total of 337 students from 59 countries were enrolled in this course between 2010 and 2012, of which 219 were awarded the certificate for the successful completion of the course—a completion rate of 65%.

In order to assess the outcome of this course, an online questionnaire was designed and pilot tested in order to assess if the questionnaire was interpreted the same by everyone in the study. While keeping research capacity strengthening as the primary focus, the following additional parameters were also taken into consideration while developing the questionnaire: the use of the knowledge acquired by professionals in their practice as well as their teaching and publication abilities, which was measured by the number of publications after their training. Moreover, we wanted to assess whether the course had influenced their promotions to higher or more important positions.

In early 2014, the online questionnaire was sent to all 219 graduates (2010–2012) of the Sexual and Reproductive Health and Research course. A total of 175 students from 45 countries participated in the study by answering the online questionnaire, showing a response rate of 80%.

### Ethical consideration

This paper is the evaluation of the impact of a training course on research methodology provided to health professionals who reside in 59 different countries and who took part in the 8-month training in 2010, 2011, or 2012. With respect to the ethical considerations and freedom of course participants, we did not want to send reminders to the participants.

Participants who had taken the course were asked to complete a questionnaire. The Executive Committee and the review board of the Geneva Foundation for Medical Education and Research specifically approved the conduct of this survey.

The purpose of the evaluation was explained to the participants in e-mail, and recipients were free to accept or decline responding to the questionnaire. All participants received the explanatory e-mail, regardless of their professional background, sex, or country of residence. The questionnaire was in no way prejudiced or denigrating to the participants’ character. Furthermore, no coercion was used to influence participants’ decisions to engage in the completion of the questionnaire.

## Results

### Demographics of respondents: sex, age, and profession

Of the 175 respondents, 56% were male and 44% were female (Table 1). More than three quarters of participants (80%) were between the ages of 31 and 50, the working-age population for whom this course is likely to be most beneficial.

**Table 1:** Age and sex distribution of participants

Age group (years)	Female	Male	Total
20–30	4 (5%)	17 (17%)	21 (12%)
31–40	37 (48%)	48 (49%)	85 (49%)
41–50	26 (34%)	28 (29%)	54 (31%)
51–60	8 (10%)	1 (1%)	9 (5%)
61–70	2 (3%)	4 (4%)	6 (3%)
Total	77 (100%)	98 (100%)	175 (100%)

We did not find any difference in terms of age, sex, and qualifications of the 20% of participants who did not respond to this questionnaire.

Similarly, a majority of the respondents (70%) were either medical doctors (61%) or nurse/midwives (9%), while 10% were public health professionals with a non-medical background such as economics, anthropology, or sociology (Table 2).

**Table 2:** Participants by professional qualification

Participants’ professional qualification	Number (%)
Medical degree	106 (61%)
Public health	17 (10%)
Midwife	4 (2%)
Nurse	13 (7%)
Other	35 (20%)
Total	175 (100%)

**Fig. 1:**
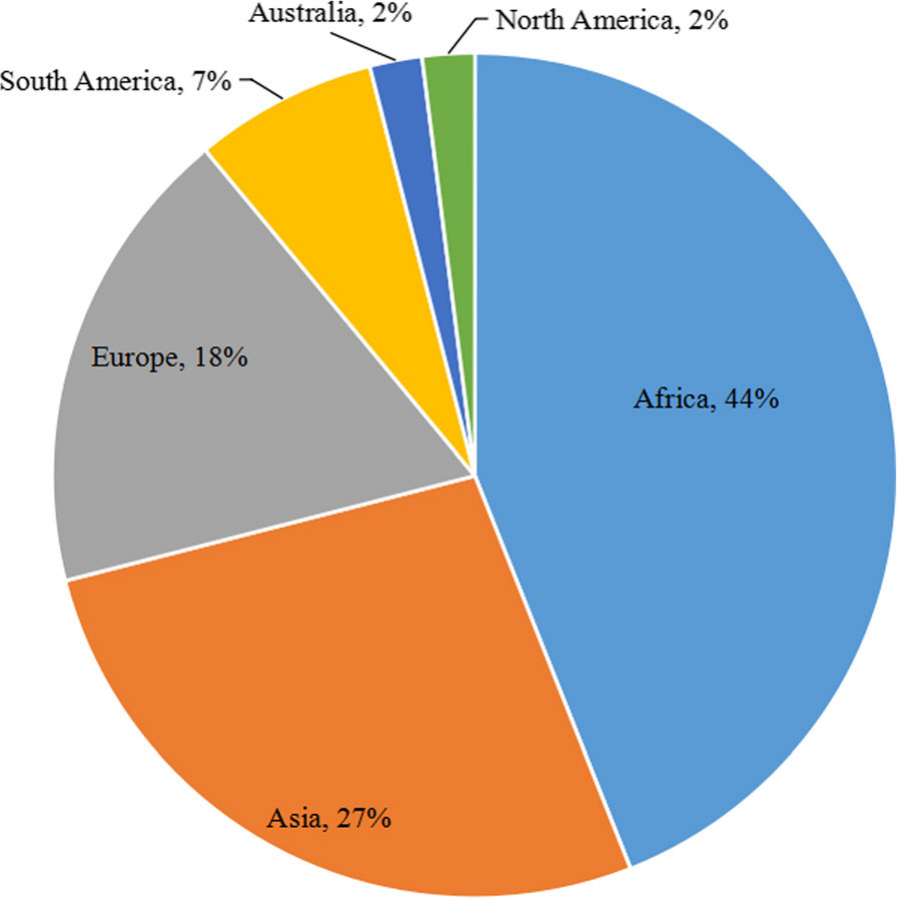
Course participants’ distribution by region

The participants of this study reside in 45 different countries from six continents. The majority live in Africa (44%) and Asia (27%) while others are from Europe (18%), South America (7%), Australia (2%), and North America (2%). Figure 1 represents the diversity of the GFMER online course participants.

The administered questionnaire highlights the views of the study participants regarding the impact of the course on their professional careers and skills (Table 3). The emphasis on the importance of research to these professionals was substantiated through the majority involvement in research development and implementation. Only a few of them (9%) had no intentions of conducting further research on their chosen topic. The research topics are selected by the health professionals themselves and are relevant to their professional activities and practice, correlating to the rate of successful implementation of research, the active involvement in scientific writing, and inclination towards teaching (47%).

**Table 3:** Participants’ feedback on the impact of the course to their professional careers and skills

	Participants’ feedback (%)		
	Yes	No	Not applicable
1. a. Have you been able to apply the knowledge and skills learned during the course? b. Have you been involved in teaching?	171 (98%) 118 (67%)	4 (2%) 57 (33%)	0 (0%) 0 (0%)
2. Since completion of the GFMER course, have you implemented the research project that you designed during the course?	69 (39%)	106 (61%)	0 (0%)
3. Have you been involved in designing or implementing a research project?	129 (74%)	46 (26%)	0 (0%)
4. Have you published a scientific article as author or co-author?	82 (47%)	93 (53%)	0 (0%)
5. Since taking the course, have you had a promotion in your professional career?	81 (46%)	94 (54%)	0 (0%)
6. Do you think that this course has helped advance your career (i.e. promotion)?	142 (81%)	33 (19%)	0 (0%)

The 47% of respondents who had authored or co-authored a scientific paper were asked to provide the complete bibliographic information of the articles. Of the articles authored or co-authored by the respondents, 49% were published in regional and international journals, such as the Journal of Research in Medical Science; Journal of Perinatology; International Breastfeeding Journal; Journal of Nursing & Care; Journal of Research in Behavioural Science; Journal of Pathology and Microbiology, Contraception, Obstetric and Gynaecology; Australian Medical Journal; Tropical Doctors; International Journal of Infectious Diseases; Pan African Journal; and others.

### Projects designed and/or implemented by graduates

**Fig. 2:**
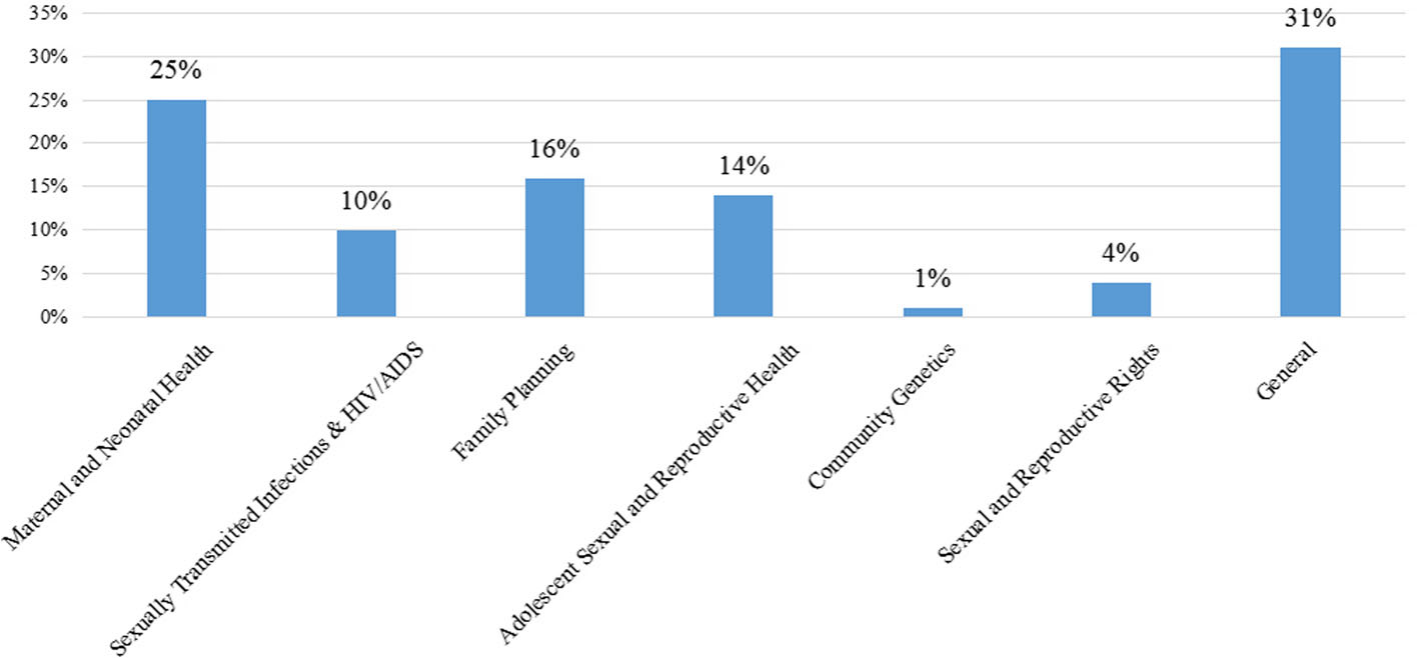
Research projects implemented or designed by course participants

**Fig. 3:**
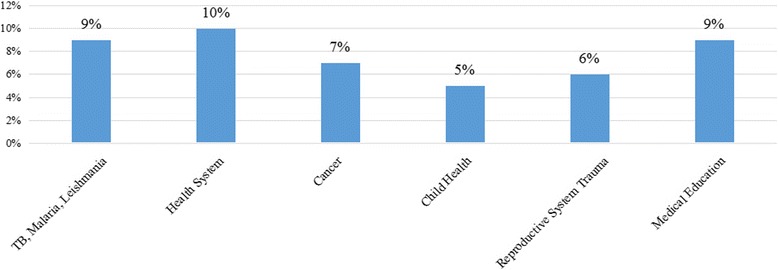
The graphic shows the thematic areas of projects classified as general

The themes for research projects designed or implemented by the study participants are shown in Fig. 2. The projects have been grouped into themes aligned to the module topics for the course or the broader sexual and reproductive health field. Where topics cover more than one field, they are grouped under the dominant field. For example, HIV/AIDS and adolescents is considered to be Adolescent Sexual and Reproductive Health. Those falling outside of these topics, e.g. health system research, reproductive cancer, or sexual health, are categorized as “General”, representing 31% of these research projects (Fig. 3). Other fields include Maternal and Neonatal Health (25%), Family Planning (16%), Adolescent Sexual and Reproductive Health (14%), Sexually Transmitted Infections and HIV/AIDS (10%), Sexual and Reproductive Rights (4%), and Community Genetics (1%).

Finally, over 8 in 10 respondents said that the course had helped them advance in their careers.

## Discussion

The relevance of the findings of this study to international audiences from a theoretical point of view is based on the “interactivity” as one of the key components that makes the online approach to capacity building effective. The interactivity has been shaped in the article as the ongoing interaction between the target audiences/students and the pedagogical group (all stockholders particularly personal coach assigned to each learner) with the aim to adapt the course to the needs of students. This concept has been well evaluated and proven for the “classical” face-to-face training. The GFMER course eloquently proved this theoretical concept as important, applicable, and effective for the online, e-learning platform.

In regard to the objectives of the course, the surveys reveal a favourable outcome. Nearly all respondents (98%) stated that the knowledge and understanding acquired during the course had been applied to their professional work. This is also reflected in the fact that 39% have implemented the research project designed specifically for the course while 74% were involved in some form of research. A major aim of the course is to encourage a deeper understanding and involvement in scientific writing, and it is laudable that 47% of respondents have authored or co-authored a scientific article. Overall, 81% believe that this course has helped advance their career.

Students’ attrition is a substantial challenge in e-learning. Different studies report attrition rates from 20 to 80% [6]. In order to reduce the course attrition, all the information about the course structure (i.e. list of course modules, module calendars, contents of the modules, assignments, and other teaching materials) is provided in a very short and simple text to the participants. Furthermore, a coach is assigned to each student as the primary contact. Between 2010 and 2012, 219 students out of 337 who had enrolled in the course successfully completed it. This gives a dropout rate of 35%, which, in reference to the current data and existing literature on online course attrition, is a fairly low attrition rate.

However, our study has some limitations. Firstly, the main outcome of interest of our study was its research capacity strengthening, e.g. number of research projects designed and implemented by course participants and number of scientific papers they published. The study does not measure the effect of the course on changes in public health or clinical practices or on public health policy and programme outcomes. This could be the subject of further studies. Secondly, while GFMER received feedback from 80% of students, this leaves 20% unaccounted for with regard to the stated objectives of the course and these student responses may have provided even more clarity on the outcomes of the study. Finally, as can be seen from the findings, two thirds of the respondents were involved in teaching and almost half of the course participants reported a promotion. The course could have contributed to the promotions although it is difficult to determine if the course was the main trigger for the promotion. The promotion could independently be making it easier to cascade the knowledge gained or spearhead implementation of research projects or both.

## Conclusions

The GFMER/WHO course offers healthcare workers up-to-date training in sexual and reproductive health research and methodology through various interactive methods of learning and does so in partnership with the World Health Organization, universities, and other organizations around the world. Participants are offered a structured course tailored to meet their needs and a straightforward curriculum. Efforts have been put in place to decrease the rate of attrition. Furthermore, participants are given the opportunity to continue their relationship with GFMER through membership with the Foundation. In this way, the course contributes to global efforts in research capacity strengthening of health professionals involved in sexual and reproductive health.

Because most of the participants are in the working-age group and come from the low- and middle-income countries, mainly Africa and Asia where maternal mortality and morbidity indices are the highest, alongside fragile health systems, the target audience for the course was therefore appropriate.

Through years of active involvement in the provision of education in the field of sexual and reproductive health research, and through online courses, the Foundation has built an international network of health professionals. GFMER and partners, particularly WHO, will constantly revise and update the course curricula to adapt them to the needs based on the reality of the field and students’ feedback.

GFMER and its partners can view the findings of this study with some satisfaction. We conclude that our methodology of easy accessibility of training material and a high level of interactivity with course tutors including involvement of local coaches who have previously completed the course is a scalable model that can be used in many online courses. The Foundation’s long track record in education on sexual and reproductive health research and willingness to seek and learn from the feedback from students helped us to better understand learner needs and how to use evolving opportunities in order to develop better and more targeted training materials for a diverse audience.
